# Analysis of single nucleotide variants of *HFE* gene and association to survival in The Cancer Genome Atlas GBM data

**DOI:** 10.1371/journal.pone.0174778

**Published:** 2017-03-30

**Authors:** Sang Y. Lee, Junjia Zhu, Anna C. Salzberg, Bo Zhang, Dajiang J. Liu, Joshua E. Muscat, Sara T. Langan, James R. Connor

**Affiliations:** 1 Department of Neurosurgery, The Pennsylvania State University College of Medicine, Penn State Hershey Medical Center, Hershey, Pennsylvania, United States of America; 2 Department of Public Health Sciences, The Pennsylvania State University College of Medicine, Penn State Hershey Medical Center, Hershey, Pennsylvania, United States of America; 3 Institute for Personalized Medicine, The Pennsylvania State University College of Medicine, Penn State Hershey Medical Center, Hershey, Pennsylvania, United States of America; 4 Department of Biochemistry & Molecular Biology, The Pennsylvania State University College of Medicine, Penn State Hershey Medical Center, Hershey, Pennsylvania, United States of America; 5 Department of Public Health Sciences and Biochemistry & Molecular Biology, Institute for Personalized Medicine, The Pennsylvania State University College of Medicine, Penn State Hershey Medical Center, Hershey, Pennsylvania, United States of America; University of Cincinnati College of Medicine, UNITED STATES

## Abstract

Human hemochromatosis protein (HFE) is involved in iron metabolism. Two major HFE polymorphisms, H63D and C282Y, have been associated with an increased risk of cancers. Previously, we reported decreased gender effects in overall survival based on H63D or C282Y HFE polymorphisms patients with glioblastoma multiforme (GBM). However, the effect of other single nucleotide variation (SNV) in the *HFE* gene on the cancer development and progression has not been systematically studied. To expand our finding in a larger sample, and to identify other *HFE* SNV, we analyzed the frequency of somatic SNV in *HFE* gene and its relationship to survival in GBM patients using The Cancer Genome Atlas (TCGA) GBM (Caucasian only) database. We found 9 SNVs with increased frequency in blood normal of TCGA GBM patients compared to the 1000Genome. Among 9 SNVs, 7 SNVs were located in the intron and 2 SNVs (i.e., H63D, C282Y) in the exon of *HFE* gene. The statistical analysis demonstrated that blood normal samples of TCGA GBM have more H63D (p = 0.0002, 95% Confidence interval (CI): 0.2119–0.3223) or C282Y (p = 0.0129, 95% CI: 0.0474–0.1159) HFE polymorphisms than 1000Genome. The Kaplan-Meier survival curve for the 264 GBM samples revealed no difference between wild type (WT) HFE and H63D, and WT HFE and C282Y GBM patients. In addition, there was no difference in the survival of male/female GBM patients based on HFE genotype. There was no correlation between HFE expression and survival. In conclusion, the current results suggest that somatic HFE polymorphisms do not impact GBM patients’ survival in the TCGA data set of GBM.

## Introduction

Uptake of iron is regulated by several proteins including HFE, the hemochromatosis protein. HFE is a major histocompatibility complex class 1 protein thought to play a key role in the regulation of body iron. There are two major mutation sites in the gene for *HFE* [1]: a single mutation of C to G at nucleotide 187 results in substitution of aspartic acid for histidine at amino acid 63 (H63D), and a second of G to A at nucleotide 845, results in substitution of tyrosine for cysteine at amino acid 282 (C282Y). Polymorphisms in the *HFE* gene are relatively common in Caucasians with the frequency for H63D HFE heterozygote and homozygote around 22–28% (22–24% heterozygote and 2.4–4% homozygote) and for C282Y HFE heterozygote and homozygote about 9–10% (8–10% heterozygote and 0.4–1% homozygote) [2–4]. Both HFE polymorphisms are associated with increased cellular iron uptake [5–7] which may indicate an increased need for iron for cancer cell proliferation. The increase in intracellular iron may also create an environment for DNA damage that could transform a cell into a tumor cell. Expression of H63D or C282Y is more frequent in patients in a number of cancers including malignant glioma compared to unaffected controls [8–13]. Results from a HFE and brain tumor study demonstrated a higher frequency of H63D HFE polymorphism in patients with malignant gliomas [8]. Multiple lines of evidence also suggest that, in addition to being a risk factor for cancer, expression of C282Y HFE polymorphism may enhance its progression [14–16]. Indeed, in cell culture, both neuroblastoma and a number of astrocytoma cell lines show evidence of Temodar (chemotherapy standard of care) and radiation resistance [17].

Malignant gliomas, the most common and aggressive type of primary brain tumors, have a high mortality rate. Uncontrolled proliferation and enhanced survival under ischemic conditions are cancer cell hallmarks. Cancer cells require intracellular metabolites including iron to fuel the active metabolism required for their rapid proliferation [18, 19]. H63D and C282Y HFE polymorphisms are associated with an increased risk of cancers [20, 21]. However, the association between gene variations in iron metabolism genes and survival of malignant gliomas such as glioblastoma multiforme (GBM) has not been examined. Previously, in human studies, we found that male GBM patients that expressed H63D HFE polymorphism had poorer survival rates than male GBM patients expressing wild type (WT) HFE [22]. Of those GBM patients expressing C282Y HFE, female patients had lower overall survival compared to males [22]. It should be noted, however, the sample number in that study was small. Therefore, we sought to confirm those findings in a larger sample size study in the present study. The effect of other single nucleotide variant (SNV) in the *HFE* gene on cancer development and progression has not been systematically studied. Therefore, we also analyzed SNVs in the *HFE* gene using The Cancer Genome Atlas (TCGA) GBM database to determine the risk of malignancy in human GBM. Here, we report our findings on the altered SNVs in blood normal of TCGA GBM with a control group and the association between HFE genotype and overall survival of GBM patients. We also studied whether there is an association between HFE polymorphisms and survival of TCGA GBM patients; and, if so, whether that association is dependent on gender and/or *HFE* gene expression level.

## Methods

### Download of *HFE* gene variants, *HFE* gene expression, and patient outcomes of the TCGA GBM patients

The TCGA contains various datasets for GBM and adjacent normal tissue samples, accessible via its TCGA data portal [23]. However, due to lack of Variant Call Format (VCF) or Mutation Annotation Format (MAF) files of GBM in the portal (personal communication with TCGA), gene sequence data was obtained to identify SNVs in the *HFE* gene of the GBM patients. The sequence data are accessible via the National Cancer Institute’s Cancer Genomics Hub (CGHub); and, can be downloaded using the GeneTorrent software. However, the download process for the whole genome sequences requires extensive computing resources, e.g. network bandwidth and disk space (several TB for 528 samples). Therefore, we used the GTFuse software (AnnaiSystems, Carlsbad, CA), which allowed us to extract and download only the regions of interest from the complete mapped sequence (BAM) files. Variants in the *HFE* gene were then identified from these sequences using the Genome Analysis Toolkit (GATK) software based on the GATK best practices pipeline [24]. The University of California Santa Cruz (UCSC) table browser was used to download all of the variants of the *HFE* gene to determine allele frequency of the general population based on dbSNP Build 142. For comparison, we also downloaded the frequency of SNVs in the *HFE* gene of 1000Genome using VCF file from The International Genome Sample Resource. We then queried it through tabix. Clinical data such as gender, age, race, ethnicity, histological type, survival and outcome, and *HFE* gene expression level were downloaded from the TCGA data portal. Furthermore, the outcome data file was used to identify the race of each patient. The sequence and clinical information were linked via patients’ TCGA IDs. In this study, we focused on the role of SNVs in the *HFE* gene and its association with survival in Caucasian patient samples as HFE polymorphisms are most prevalent in the Caucasian population.

### Statistical analysis

We selected only Caucasian samples with both SNVs in the *HFE* gene and clinical information available for gender and survival analysis. The proportions of homozygote or heterozygote variations at each position were calculated and their exact 95% confidence intervals were generated. The proportions were then compared to the reference proportions from the 1000Genomes project (when available) using exact binomial test. Additionally, the associations between survival and *HFE* gene mutation/gender/1 year survival status were examined using Fisher‘s exact test. Kaplan-Meier plots were used to illustrate the relationship between patient’s overall survival and HFE polymorphisms; and, these relationships were tested by log-rank test. The difference between *HFE* gene expression and HFE genotype was analyzed by two sample t-test. All analyses were performed using R program language version 3.2.1 (R Foundations); and, the statistical significance was 0.05.

## Results

### Characterization of primary Tumor Patient (TP) and Blood Normal (NB) samples of GBM patients in TCGA database

HFE polymorphisms are more prevalent in Caucasians than in other races, thus we only used Caucasians data for the frequency of SNVs including H63D and C282Y HFE polymorphisms in the *HFE* gene and its association with survival analysis. There were 340 total samples for SNV data, including HFE genotype, available in primary tumor patient (TP) samples in the TCGA GBM database. Among the 340 samples, 11 samples had duplicate records. Thus, the total number of samples for SNV and HFE genotype in TP sample was 329. The total number of patients who had clinical information was 511. In the merged dataset (genotype + clinical data) there were a total of 296 TP samples: 264 Caucasian, 6 Asian, 18 Black, and 8 unknown. Of 264 Caucasian primary tumor GBM samples, there were 167 male and 97 female (Table 1). Meanwhile, the total number of samples for HFE genotype in blood normal (NB) sample was 332. Among them, 291 samples have both genotype and clinical data. These consisted of 261 Caucasian, 5 Asian, 18 Black, and 7 unknown. The age for the TP sample group ranged from 21 to 89 years old. The median age of male TP was 61 years old; and, the median age of female TP was 63 years old. Among TP and NB samples that share same patient ID, two samples’ HFE genotype was different among NB and TP samples. One patient’s NB sample had C282Y heterozygote, but its tumor tissue had no mutation at 282 amino acid in the *HFE* gene. The other NB sample also had C282Y heterozygote; while, the TP sample had C282Y homozygote.

**Table 1:** Characteristics between our previous study, TCGA GBM, and 1000Genome^1^.

	PSHCI GBM (n = 97)^2^	TCGA GBM_TP (n = 264)	TCGA GBM_NB (n = 261)	1000Genome Phase 3 (n = 185)^3^
Median age (years old)	64, (Male: 60; Female: 67)	62, (Male: 61; Female: 63)	62, (Male: 61; Female: 63)	NA
Range of age (years old)	24–88, (Male: 24–78; Female: 27–88)	21–89, (Male: 23–89; Female: 21–85)	21–89, (Male: 23–89; Female: 21–85)	NA
Male: Female (ratio)	49: 48 (1.02: 1)	167: 97 (1.72: 1)	164: 97 (1.69: 1)	80: 102 (1: 1.28)

^1^ The studied samples for our previous study, TCGA GBM and 1000Genome were all Caucasians

^2^ Lee SY, et al. J Neurooncol. 2015;122:97–104.

^3^ There were a total of 1,077 samples (527 male, 550 female) listed on the website, however, only a subset have sequences. There were 185 European subpopulation (80 male, 102 female, 3 unknown). Age information was not available.

### Identification of altered Single Nucleotide Variant (SNV) in the *HFE* gene of TCGA GBM

To determine which SNVs were altered in GBM, we evaluated the frequency of SNVs in NB in TCGA GBM; and, we then compared that frequency with the frequency in 1000Genome (genome sequence data of at least 1000 anonymous participants). We found 9 SNVs in the *HFE* gene which have increased frequency in blood normal compared to the 1000Genome (Table 2). Among those 9 SNVs, 7 SNVs were located in the intron and 2 SNVs (i.e., H63D, C282Y) were in the exon of *HFE* gene. The statistical analysis indicated that blood normal of TCGA GBM have more H63D (p = 0.0002, 95% Confidence interval (CI): 0.2119–0.3223) or C282Y (p = 0.0129, 95% CI: 0.0474–0.1159) HFE polymorphisms than 1000Genome (Table 2). The H63D genotype was present in 26.4% (23.0% heterozygote + 2.3% homozygote + 1.1% compound mutation—a sample with both H63D and C282Y HFE polymorphisms); while, the C282Y genotype was present in 7.6% (6.1% heterozygote + 0.4% homozygote + 1.1% compound mutation) in blood normal samples (Table 3). When we stratified H63D and C282Y HFE genotype by gender (Table 4), we found 23.8% H63D heterozygote, 2.4% H63D homozygote, and 1.2% compound mutation in male. In female, there were 21.6% heterozygote, 2.1% homozygote, and 1% compound mutation for H63D *HFE*. For the C282Y genotype, there were 5.5% C282Y heterozygote, 1.0% C282Y homozygote, and 1.2% compound mutation in male. There were 7.2% C282Y heterozygote, no C282Y homozygote, and 1% compound mutation in female. When we analyzed gender effect on H63D and C282Y HFE genotype between our previous sample and TCGA GBM NB samples, there were no differences between male and female in the H63D genotype (p = 0.31 for male & p = 0.86 for female by Chi-Square test) or in the C282Y genotype (p = 0.68 for male & p = 0.98 for female by Chi-Square test).

**Table 2:** List of increased frequency of Single Nucleotide Variant (SNV) between TCGA GBM NB sample and 1000Genome^1^.

POS^2^	dbSNP	REF^3^	ALT^4^	1000GenomesPhase3_Info (EUR_AF)	No_mut #	Mut^5^ #	Total #	Nucleotide change	Amino acid change	P value	95% CI^6^ (low)	95% CI (high)
**26093236**	rs1800758	G	A	0.0994	184	77	261	c. 892+48G>A		<0.0001	0.2404	0.3544
**26094367**	rs1572982	G	A	0.4722	73	188	261	c. 1007-47G>A		<0.0001	0.6616	0.7739
**26091336**	rs2071303	T	C	0.3658	121	140	261	c. 340+4T>C		<0.0001	0.4739	0.5981
**26091179**	rs1799945	C	G	0.172	192	69	261	c. 187C>G	His63Asp	0.0002	0.2119	0.3223
**26087856**	rs2858993	T	A	0.3976	120	117	237	c. 76+112T>A		0.0028	0.4283	0.5592
**26090381**	rs62396165	C	G	0.1074	37	12	49	c. 77-688C>G		0.0048	0.1334	0.3887
**26088407**	rs3799374	A	G	0.1113	31	11	42	c. 76+663A>G		0.0052	0.1386	0.4204
**26093141**	rs1800562	G	A	0.0427	241	20	261	c. 845G>A	Cys282Tyr	0.0129	0.0474	0.1159
**26093297**	rs2794717	G	A	0.007	256	5	261	c. 893-50G>A		0.0378	0.0062	0.0441

^1^ The studied samples were all Caucasians

^**2**^POS: Position

^**3**^REF: Reference

^**4**^ALT: Alternative

^**5**^Mut: Mutation

^**6**^CI: Confidence interval

**Table 3:** Frequency of HFE genotype in our previous study, TCGA GBM (TP, NB) samples^1^.

	PSHCI GBM (n = 97)^3^	TP (n = 264)	NB (n = 261)
**H63D/+**	18 (18.6%)	61 (23.1%)	60 (23.0%)
**H63D/H63D**	1 (1.0%)	6 (2.3%)	6 (2.3%)
**C282Y/+**	6 (6.2%)	15 (5.7%)	16 (6.1%)
**C282Y/C282Y**	0 (0.0%)	2 (0.8%)	1 (0.4%)
**H63D/C282Y**	0 (0.0%)	3 (1.1%)	3 (1.1%)
**+/+** ^2^	72 (74.2%)	177 (67.0%)	175 (67.0%)
**Total**	97	264	261

^1^ The studied samples of PSHCI and TCGA GBM patients were all Caucasians

^2^ +/+: no mutation at 63 and 282 amino acids of *HFE* gene

^3^ Lee SY, et al. J Neurooncol. 2015;122:97–104.

**Table 4:** Allele and genotype frequencies of H63D and C282Y HFE polymorphisms in our previous study^1^ and TCGA GBM (TP, NB) samples^1^, stratified by gender.

	PSHCI GBM (n = 97)^2^		TP (n = 264)		NB (n = 261)	
	Male (n = 49)	Female (n = 48)	Male (n = 167)	Female (n = 97)	Male (n = 164)	Female (n = 97)
**Genotype**						
H63D/+	9 (18.4%)	9 (18.8%)	40 (24.0%)	21 (21.6%)	39 (23.8%)	21 (21.6%)
H63D/H63D	0 (0.0%)	1 (2.1%)	4 (2.4%)	2 (2.1%)	4 (2.4%)	2 (2.1%)
C282Y/+	2 (4.1%)	4 (8.3%)	9 (5.4%)	6 (6.2%)	9 (5.5%)	7 (7.2%)
C282Y/C282Y	0 (0.0%)	0 (0.0%)	2 (1.2%)	0 (0.0%)	1 (0.6%)	0 (0.0%)
H63D/C282Y	0 (0.0%)	0 (0.0%)	2 (1.2%)	1 (1.0%)	2 (1.2%)	1 (1.0%)
+/+	38 (77.6%)	34 (70.8%)	110 (65.9%)	67 (69.1%)	109 (66.5%)	66 (68.0%)
**Alleles**						
H63D	9/98 (9.2%)	11/96 (11.5%)	50/334 (15.0%)	26/194 (13.4%)	49/328 (14.9%)	26/194 (13.4%)
C282Y	2/98 (2.0%)	4/96 (4.2%)	15/334 (4.5%)	7/194 (3.6%)	13/328 (4.0%)	8/194 (4.1%)

^1^ Caucasian only, Values were expressed as n = N (%) or n = N

^2^ Lee SY, et al. J Neurooncol. 2015; 122: 97–104.

The frequency of other SNVs in the *HFE* gene, i.e. S65C and Q277K, in NB was not different than the frequency in 1000Genome. A small number of NB samples demonstrated variation at that position, corresponding to S65C and Q277K. There were 6 samples with variations in S65C and only one sample had variation in Q277K in NB samples of TCGA GBM.

Interestingly, one intron position SNV (Position 26091336), a known benign risk factor for hemochromatosis and located near exon 2, has increased frequency in NB samples compared to the frequency of 1000Genome (p<0.0001, 95% CI: 0.4739–0.5981) (Table 2).

Next, we compared the SNV of TP samples with the SNV of NB samples to study whether tumor tissues demonstrate somatic mutation. There was an increased ratio at 5 SNVs (all intron positions of *HFE* gene) in tumor samples compared to the blood normal samples. However, that was not statistically significant.

### Association between HFE genotype and patient survival in primary tumor tissue patients of TCGA GBM data

**Fig 1:**
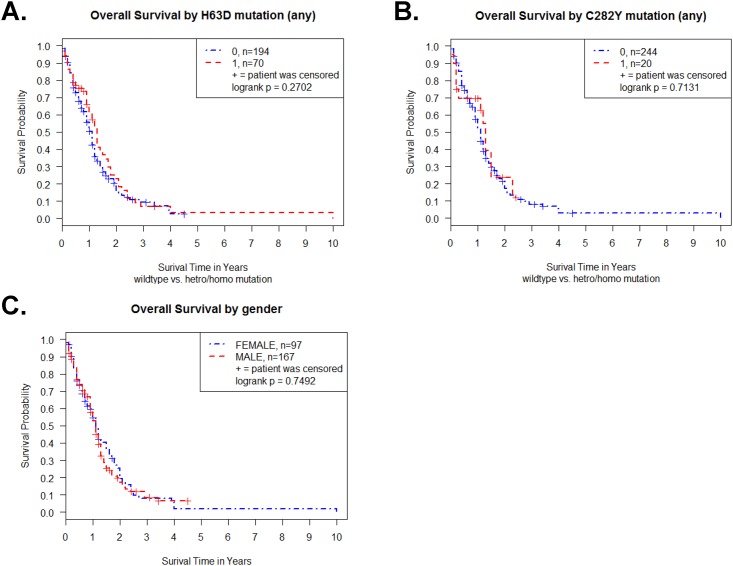
Kaplan-Meier survival curve for TCGA GBM patients (n = 264) with HFE genotype and gender. (A) Survival curve of GBM patients with H63D HFE polymorphism. (B) Survival curve of GBM patients with C282Y HFE polymorphism. (C) Survival curve of male and female GBM patients. Plot symbols indicate censored (missing data) points. Statistical analysis was performed by log-rank test and indicated as p value.

The Kaplan-Meier survival curve for the 264 Caucasian GBM samples revealed no difference between wild type (WT) and H63D HFE polymorphism GBM samples in log-rank test (p = 0.27) (Fig 1A). There was no survival difference between WT and C282Y HFE polymorphism GBM samples in log-rank test (p = 0.71) (Fig 1B).

Malignant gliomas occur more frequently in males than females, thus we then tested a gender effect on survival analysis [25]. The survival between male (n = 167) and female (n = 97) GBM patients was not different in Caucasian GBM tumor samples (p = 0.75 in log-rank test) (Fig 1C).

**Fig 2:**
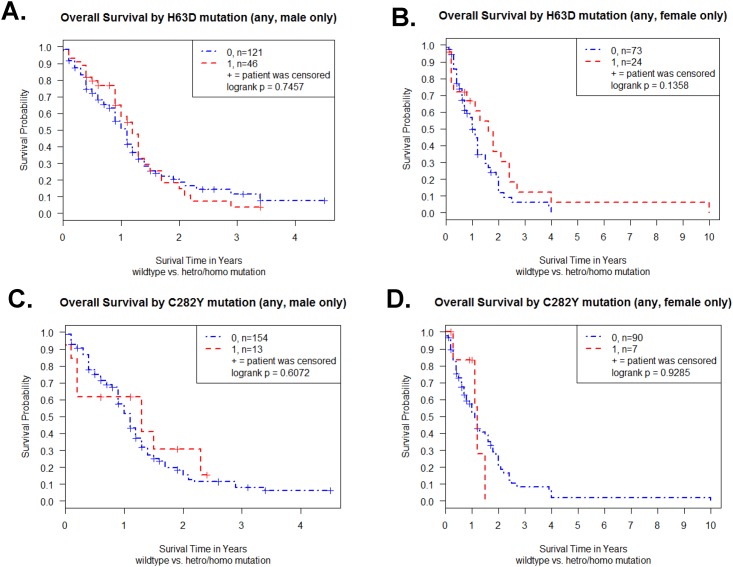
Effect of gender on Kaplan-Meier survival curve of TCGA GBM patients. (A) Survival curve of male GBM patients (n = 167) with or without H63D HFE polymorphism. (B) Survival curve of female GBM patients (n = 97) with or without H63D HFE polymorphism. (C) Survival curve of male GBM patients (n = 167) with or without C282Y HFE polymorphism. (D) Survival curve of female GBM patients (n = 97) with or without C282Y HFE polymorphism. Plot symbols indicate censored points. Statistical analysis was performed by log-rank test and indicated as p value. Patients with either H63D or C282Y HFE polymorphism were represented in the figures by the red line and those without were represented by the blue line.

Previously, we reported that male H63D HFE polymorphism GBM patients had poorer survival than male WT HFE GBM patients; and, female C282Y HFE polymorphism demonstrated poorer survival than male C282Y HFE polymorphism patients [22]. Therefore, we determined the relationship between HFE genotype and overall survival to be further stratified by gender. The Kaplan-Meier survival curve for the 167 male GBM patients revealed no difference between WT and H63D HFE polymorphism (p = 0.75 in log-rank test) (Fig 2A). The Kaplan-Meier survival curve for the 97 female GBM patients revealed no difference between WT and H63D HFE polymorphism (p = 0.14 in log-rank test) (Fig 2B). Furthermore, when examining C282Y HFE polymorphism and survival analysis, there were no differences between WT and C282Y HFE polymorphism in males (p = 0.61 in log-rank test) as well as in females (p = 0.93 in log-rank test) (Fig 2C & 2D).

**Fig 3:**
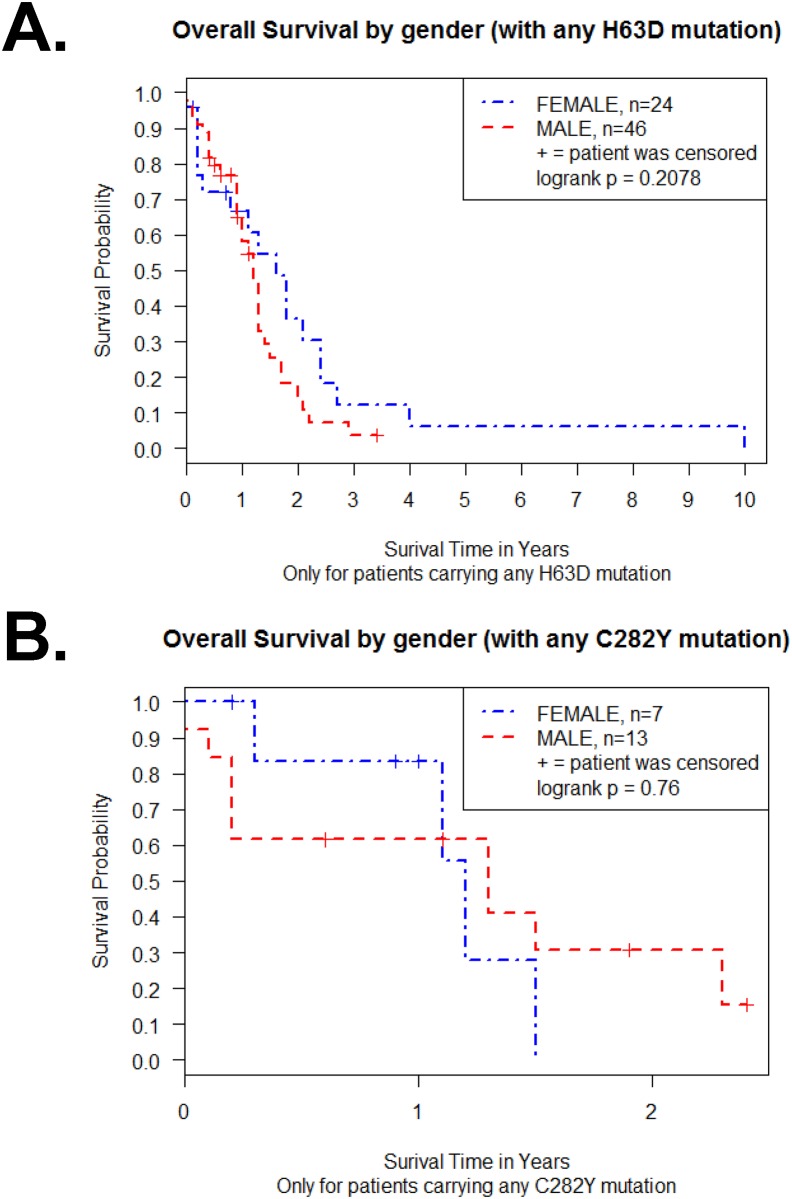
Effect of gender on Kaplan-Meier survival curve of H63D or C282Y HFE polymorphism GBM patients. (A) Survival curve of male and female H63D HFE polymorphism GBM patients (n = 70). (B) Survival curve of male and female C282Y HFE polymorphism GBM patients (n = 20). Plot symbols indicated censored points. Statistical analysis was performed by log-rank test and indicated as p value.

Next, we determined survival between males and females with H63D or C282Y HFE polymorphisms. The Kaplan-Meier survival curve for the 70 H63D GBM patients revealed no difference between male and female patients (p = 0.21 in log-rank test) (Fig 3A). The Kaplan-Meier survival curve for the 20 C282Y GBM patients revealed no difference between male and female patients (p = 0.76 in log-rank test) (Fig 3B). Next, because it is known that median survival of GBM is less than a year, we analyzed whether 1 year survival status is affected by HFE genotype. We found one year survival status was not affected by H63D mutation (p = 0.31) or C282Y mutation (p = 0.58) in Fisher‘s exact test.

**Fig 4:**
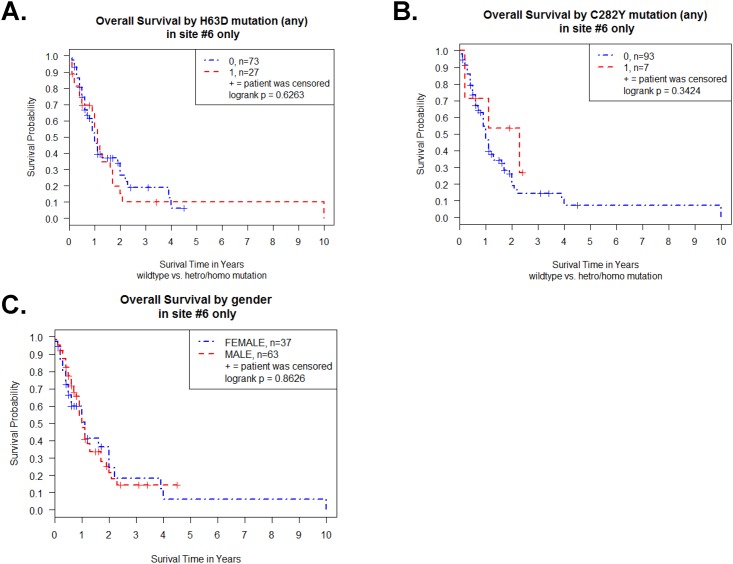
Kaplan-Meier survival curve for TCGA GBM patients with HFE genotype and gender in a single site samples (n = 100). (A) Survival curve of GBM patients with H63D HFE polymorphism. (B) Survival curve of GBM patients with C282Y HFE polymorphism. (C) Survival curve of male and female GBM patients. Plot symbols indicate censored points. Statistical analysis was performed by log-rank test and indicated as p value.

Moreover, we used only single sample collection site data (n = 100 at Henry Ford Hospital) from 264 GBM samples to exclude any possibility of regional differences. The site information and sample number of all 264 GBM samples are indicated in Table 5. The Kaplan-Meier survival curve for the 100 Caucasian GBM patients who enrolled at Henry Ford Hospital revealed no difference between WT HFE GBM patients and H63D HFE polymorphism GBM patients in log-rank test (p = 0.63) (Fig 4A). The survival between WT HFE GBM patients and C282Y HFE polymorphism GBM patients was also not different in log-rank test (p = 0.34) (Fig 4B). We further tested a gender and HFE genotype effect on survival of Henry Ford Hospital GBM patients’ samples. We did not observe any statistical difference (p = 0.86 in log-rank test) for survival based on gender in Henry Ford Hospital data (Fig 4C).

**Table 5:** The number of patients in each site of TCGA GBM data (total n = 264).

Site #	Center name of site	# of patients
2	MD Anderson Cancer Center	6
6	Henry Ford Hospital	100
8	UCSF	1
12	Duke	15
14	Emory University	16
15	Mayo Clinic—Rochester	1
16	Toronto Western Hospital	4
19	Case Western	22
26	University of Florida	10
27	Milan-Italy, Fondazione IRCCS Instituto Neuroligico C. Besta	17
28	Cedars Sinai	18
32	St. Joseph’s Hospital (AZ)	22
41	Christiana Healthcare	9
76	Thomas Jefferson University	20
81	CHI-Penrose Colorado	2
87	International Genomics Consortium	1

**Fig 5:**
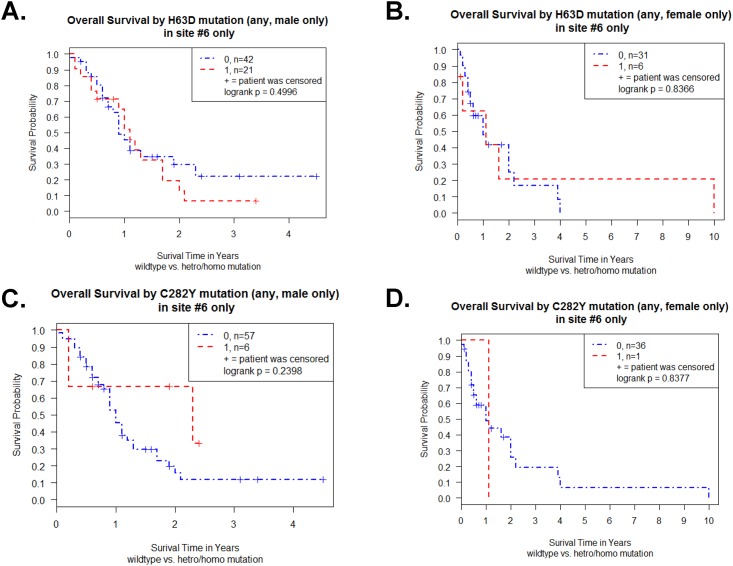
Effect of gender on Kaplan-Meier survival curve of TCGA GBM patients of a single site samples (n = 100). (A) Survival curve of male GBM patients (n = 63) with or without H63D HFE polymorphism. (B) Survival curve of female GBM patients (n = 37) with or without H63D HFE polymorphism. (C) Survival curve of male GBM patients (n = 63) with or without C282Y HFE polymorphism. (D) Survival curve of female GBM patients (n = 37) with or without C282Y HFE polymorphism. Plot symbols indicate censored points. Statistical analysis was performed by log-rank test and indicated as p value.

The Kaplan-Meier survival curve for the 63 male GBM patients revealed no difference between WT and H63D HFE polymorphism (p = 0.50 in log-rank test) (Fig 5A). The Kaplan-Meier survive curve for the 37 female GBM patients also revealed no difference between WT and H63D HFE polymorphism (p = 0.84 in log-rank test) (Fig 5B). For C282Y HFE polymorphism and survival analysis, there were no difference between WT and C282Y HFE polymorphism in male (p = 0.24 in log-rank test) (Fig 5C) as well as in female (p = 0.84 in log-rank test) (Fig 5D).

**Fig 6:**
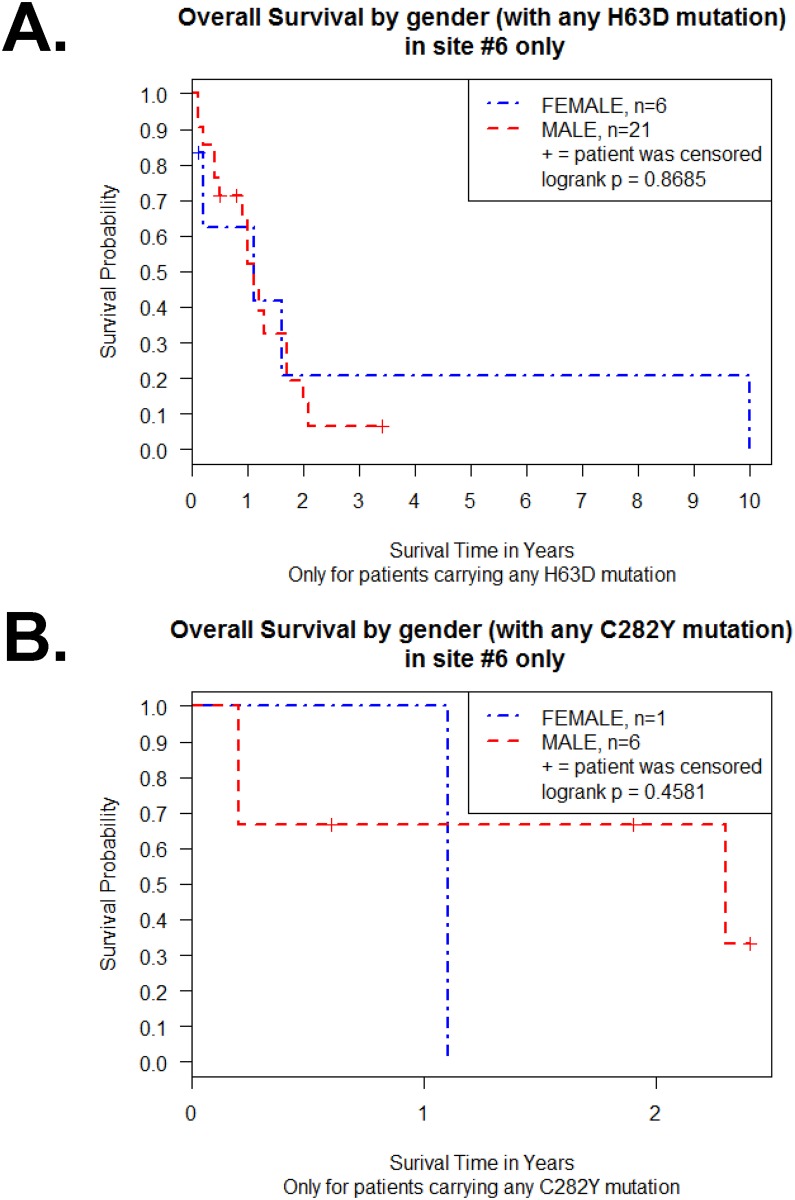
Effect of gender on Kaplan-Meier survival curve of H63D or C282Y HFE polymorphism GBM patients of a single site samples. (A) Survival curve of male and female H63D GBM patients (n = 27). (B) Survival curve of male and female C282Y GBM patients (n = 7). Plot symbols indicate censored points. Statistical analysis was performed by log-rank test and indicated as p value.

The Kaplan-Meier survival curve for the 27 H63D GBM patients revealed no difference between male and female patients (p = 0.87 in log-rank test) (Fig 6A). The Kaplan-Meier survival curve for the 7 C282Y GBM patients also revealed no difference between male and female patients (p = 0.46 in log-rank test) (Fig 6B).

### Association between the gene expression level of H63D and C282Y HFE polymorphism and patient survival in primary tumor patients of TCGA GBM data

**Fig 7:**
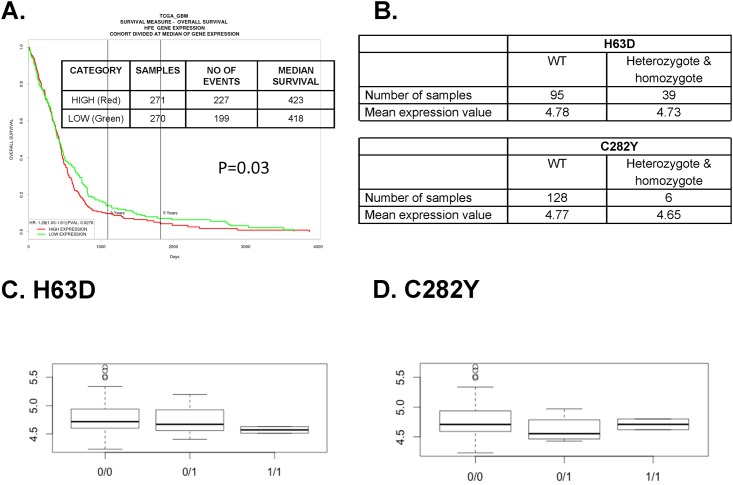
Association between H63D or C282Y HFE polymorphisms and *HFE* gene expression for survival of TCGA GBM patients. (A) Survival curve of TCGA GBM based on *HFE* gene expression. GBM patients who express high level (>50%) of *HFE* gene had poorer survival than those with low (<50%) *HFE* gene expression (p = 0.03). (B) Sample number and mean *HFE* gene expression level of TCGA GBM patients who have WT or H63D or C282Y HFE polymorphisms. The *HFE* gene expression value is the log2 of the normalized fluorescence intensity. (C) *HFE* gene expression in GBM patients who have WT or H63D HFE polymorphisms. 0/0 –no mutation for H63, 0/1 –heterozygote of H63D, 1/1 –homozygote of H63D. Y axis is *HFE* gene expression value. (D) HFE gene expression of GBM patients who have WT or C282Y HFE polymorphisms. 0/0 –no mutation for C282Y, 0/1 –heterozygote of C282Y, 1/1 –homozygote of C282Y. Y axis is *HFE* gene expression value.

According to PROGgeneV2 [26], high *HFE* gene expression in GBM patients in the TCGA database demonstrated poorer survival compared to low *HFE* gene expressed GBM patients (Fig 7A). We hypothesized that H63D and/or C282Y HFE polymorphism GBM patients express higher levels of the *HFE* gene than those GBM patients with WT *HFE* gene; and, that this difference in expression levels results in poorer survival for the H63D and/or C282Y HFE polymorphism group. Thus, we evaluated the association between HFE genotype and *HFE* gene expression (mRNA expression) level. We found a total of 134 TP samples with both HFE genotype and *HFE* gene expression data. Among them, there are 39 H63D HFE polymorphisms and 6 C282Y HFE polymorphisms (Fig 7B). The level of *HFE* gene expression was not different between the H63D HFE polymorphism GBM patients and WT patients (p = 0.31 for heterozygote, p = 0.60 for homozygote) in two sample t-test (Fig 7C). There were also no differences between C282Y HFE polymorphism and *HFE* gene expression level (p = 0.38 for heterozygote, p = 0.11 for homozygote) in two sample t-test (Fig 7D).

## Discussion

In the present TCGA GBM database study, we found that the frequency of two common HFE polymorphisms (H63D, C282Y) and several SNVs in the intron of *HFE* is increased in blood normal of TCGA GBM compared to the frequency of SNV in1000Genome. In addition, there was no difference in survival between WT HFE and H63D or C282Y HFE polymorphism TCGA GBM patients. We found no gender effect on survival of TCGA GBM patients.

We used TCGA GBM data for this study because H63D HFE polymorphism information was not available in genome-wide association studies (GWAS) of GBM patients. We utilized TCGA GBM data to analyze SNVs found in the *HFE* gene to determine cancer risk in human GBM and to further explore our previous findings of a larger sample size [22]. As in our previous study, we only used data from Caucasian samples for HFE genotype and survival analysis in TCGA GBM in this study because both HFE polymorphisms (H63D, C282Y) are more prevalent in Caucasians than in other races. The sample number of Caucasians with available SNV and clinical information in the TCGA GBM data was 264 for TP and 261 for NB. This sample number was a 2.7 fold increase compared to our previous study (97 GBM). Among our 97 GBM patients’ samples, we used 68 buccal swab samples and 29 tumor tissue samples of GBM patients for HFE genotype while all the TCGA GBM samples were used tumor tissues and blood normal for SNV analysis. Compared to our previous study, the number of H63D or C282Y HFE polymorphism GBM patients was increased from 25 (19 H63D, 6 C282Y) to 90 (70 H63D, 20 C282Y –including 3 H63D/C282Y). The number of male or female HFE polymorphism GBM samples also increased: 46 male H63D GBM samples in the present study while there were only 9 male H63D GBM samples in the previous study. Therefore, the TCGA GBM data satisfied our initial goal to increase the overall sample size.

In the present study, we found increased frequency at two exon SNVs and seven intron SNVs in the *HFE* gene of blood normal samples in TCGA GBM compared to the frequency of SNVs in 1000Genome. The two exon position SNVs were H63D and C282Y HFE polymorphisms. These data indicated H63D and C282Y HFE polymorphisms as risk factors for GBM. This difference may have been due to an uneven ratio of males to females between the study samples (1.69:1 for TCGA GBM NB, 1:1.28 for 1000Genome). There was a decreased frequency of C282Y HFE polymorphism in a population of hepatocellular carcinoma patients from Spain compared to a control group [9], while an increased frequency of H63D HFE polymorphism is noted in a sample of high glioma patients from Italy [8]. Our results indicated that the proportion of males and females in the samples may have impacted the results of statistical analysis. The proportion of males was higher in TCGA GBM samples, which may suggest that the identified SNVs are increased risk factors for male than for female GBM patients.

The impact of HFE polymorphisms on patient survival in cancers has been reported in 4 studies. Pirisi et al. found that in hepatocellular carcinoma, patients with WT HFE live longer than those with HFE polymorphisms [27] and Gannon et al. reported that patients with C282Y HFE polymorphism in epithelial ovarian cancer had decreased overall survival compared to patients with WT HFE [28]. Batschauer et al. found no association between H63D and C282Y HFE polymorphisms and WT HFE breast cancer patients’ survival in Brazilian women [29]. Recently, we reported that there was a statistically significant shorter survival for male GBM patients with the H63D HFE polymorphism compared to male GBM patients with WT HFE (p = 0.03 by log-rank test) [22]. Moreover, we reported that female GBM patients with the C282Y HFE polymorphism had decreased survival compared to male GBM patients with C282Y HFE polymorphism (p = 0.05 by log-rank test) [22]. Our previous data strongly suggest that the impact of each HFE polymorphism is distinct and dependent on the gender of the GBM patient. The different genotype effect of the HFE polymorphisms was consistent with our findings that cells similar in genetic background behave differently, with the exception of HFE type [17, 30]. However, H63D or C282Y HFE polymorphism GBM patients’ survival data of TCGA GBM database revealed no difference from WT, even when we consider gender status (Fig 3). Moreover, this lack of difference could not be attributed to population differences because when single sample collection site data was examined, we did not observe a gender effect on patient survival (Fig 6). Furthermore, the relationship between 1 year survival status and HFE genotype indicated that the proportion of H63D or C282Y HFE polymorphism was not different from WT HFE GBM patients. The study examining GBM survival between HFE genotype and *HFE* gene expression showed no association.

The samples of TCGA GBM were from 16 different locations. Among them, Henry Ford Hospital samples accounted for about one third of all TCGA GBM samples. The sample number (n = 100) of Henry Ford Hospital was similar to the study sample number from our previous study (n = 97). When we analyzed the impact of HFE genotype on GBM patient survival in one sample collection site (Henry Ford Hospital) or all combined TCGA GBM data, we did not reproduce our initial findings for gender difference. It is unclear why our findings for gender effect in our previous study were not seen in the present TCGA GBM database study. Potential reasons for the disparate findings between our previous survival data and TCGA GBM’s survival data in two common HFE polymorphisms could have been ratio of males and females, HFE genotyping method, treatment history differences (chemotherapy, immunotherapy, radiation, hormonal therapy, targeted molecular therapy), status of GBM subtype, and living area/environment of the patients, et al. As shown in Table 1, the ratio of males and females was different between studies (1.02:1 for our previous study vs. 1.72:1 for TCGA GBM). Our study samples were genotyped by PCR-restriction fragment length polymorphisms (PCR-RFLP) while the genotype of TCGA GBM was done by next generation sequencing. However, some factors mentioned above were difficult to compare between two studies. For example, our study patients and TCGA GBM patients were treated with radiation and/or multiple choice of therapies such as chemotherapy (e.g., temozolomide, irinotecan, paclitaxel, procarbazine, BCNU, CCNU, fenretinide, carboplatin, VP-16), immunotherapy (1L-13 with pseudomonas exotoxin, HSPPC-96 vaccine), targeted molecular therapy (e.g., bevacizumab, O^6^-benzylguanine), hormone therapy (dexamethasone). We also did not exclude the possibility of a different ratio of GBM subtype between our study (we don’t know our patients’ GBM subtype) and TCGA GBM. According to Verhaak et al. [31], proneural and neural subgroup GBM patients have shorter survival than classical and mesenchymal subgroup GBM patients, following aggressive treatment. Our previous study of GBM samples covered 27 counties of central Pennsylvania, many of which are medically underserved. We hypothesized that rural cancer patients have less opportunity for preventive screenings and advanced treatments compared to cancer patients living in urban environments. Recently, data show the rate of death from breast cancer decreased from 1999 to 2014 in rural Perry County by only 7 percent while the rate for all of Pennsylvania decreased by 20 percent, almost triple the rate compared to the rural group [32]. Lastly, the length of patients’ survival in our study and in TCGA GBM study was nearly the same; however, we do not exclude a possibility that our patients had a longer period between date of diagnosis and the date of treatment than that of TCGA GBM patients.

In conclusion, our data demonstrated that two common H63D and C282Y HFE polymorphisms and several SNVs in the intron of the *HFE* gene were increased in TCGA GBM; and, require further investigation into the role of cancer development and progression. Our results also demonstrated that H63D and C282Y HFE polymorphisms do not impact GBM patients’ survival in the TCGA GBM database. Further collaborative studies are needed to determine whether the role of H63D and C282Y HFE polymorphisms on cancer patients’ survival is region specific.

## Abbreviations

GBM: glioblastoma
MAF: Mutation Annotation Format
RFLP: restriction fragment length polymorphisms
SNV: single nucleotide variant
TCGA: The Cancer Genome Atlas
Tf: transferrin
TfR: transferrin receptor
VCF: Variant Call Format
WT: wild type
